# Prediction of Global Value Chain Based on Cognitive Neural Network -Take Chinese Automobile Industry as an Example

**DOI:** 10.1515/tnsci-2019-0014

**Published:** 2019-04-23

**Authors:** Qi Yang

**Affiliations:** 1 School of International Trade and Economics, ShanDong University of Finance and Economics, Jinan 250014, China

**Keywords:** Cognitive Artificial Neural Networks, Global Value Chain, Chinese Automobile Industry, Cluster, Cognitive Science

## Abstract

Cognitive artificial neural network is a hot research field which is an important part of human intelligence research. In recent years, artificial neural network has been used in the fields of neuroscience, computer science, cognitive science, mathematics and physics. With the Chinese automobile industry as the research object, the global value chain as the research tool, and the promotion of Chinese automobile industry cluster as the research objective, This paper deals with information processing by simulating neural activity in the brain according to the cognitive artificial neural network to study the upgrading of Chinese automobile industry cluster, and puts forward the related suggestion on the upgrading of Chinese automobile industry cluster. This study believes that in order to promote the upgrading of Chinese automobile industry cluster, it’s essential to promote the independent innovation of enterprises in the cluster and indispensable to strengthen government support, which has certain guiding significance to the development of Chinese automobile industry cluster.

## 1 Introduction

With the globalization of the automobile industry, the automobile industry has shifted from the traditional developed country market to the increasingly active emerging country market, and China has also taken this opportunity to integrate into the global value chain of the automobile industry. In 2007, China’s automobile production reached 8,882,400 units, an increase of 22.02% compared with 2006; sales of 8,795,500 units, an increase of 22.02% over 2006. China has become the world’s largest new car sales market. With the rapid growth of the domestic auto market, the world’s major multinational auto companies have increased their investment in China’s auto industry, and began to include wholly-owned and joint ventures in China into their global division of labor. The Chinese market has gradually become a multinational auto giant part of the “global strategy.”

As a rising country in the automobile industry, China mainly participates in the international division of labor in the automobile industry. This kind of participation has led to strong dependence on the external capital and technology of China’s automobile industry. In Sino-foreign joint ventures, multinational companies are currently providing models; multinational companies control the technical departments of joint ventures, control product support, and parts certification. Multinational corporations have obtained most of the profits of China’s automobile industry through technological control, and multinational corporations have now begun to extend the profit chain to the high-end links of parts and components, vehicle sales channels, maintenance, and financial agents. If we continue to rely on multinational corporations, China’s auto industry will gradually lose its initiative to extend to the high-end links of the value chain; at the same time, with the rise of emerging markets such as India and other labor factors that have comparative advantages, China’s auto industry will In order to maintain continued growth and survive in the fierce competition, only adjust its foreign investment policy, strengthen independent development, and realize the transformation of strategic links in the value chain.

**Figure 1:**
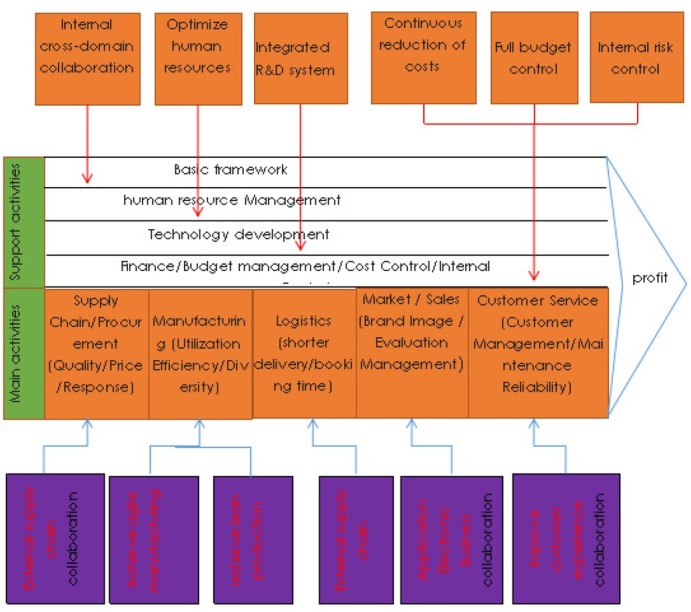
The value chain analysis module diagram for the automotive industry

In the global value chain theory system, the global industrial transfer and the world’s new division of labor pattern mainly have three kinds of driving mechanisms: producer driving, purchaser driving and intermediate driving, the industrial formation and upgrading track under different driving modes are also different, and industries will be restricted by market-oriented, hierarch-oriented, module-oriented, relationship-oriented, and leadership-oriented chain governance models in the process of upgrading along the global value chain. To study the problem of industrial upgrading from the perspective of value chain, we should not only recognize the value chain system of the industry, but also understand the chain driving force and the governance mode behind the value chain system [1]. With the development of automobile globalization, the value chain of automobile industry is an intermediate drive between producers and purchasers. Its value-added chain is bipolar, and its value is mainly distributed in the technical R & D link, production link of key parts, and sales and financial service link. Its governance model develops generally towards modules 2,3. Figure 1 shows the value chain analysis module diagram for the automotive industry.

## 2 The characteristics of Small hydropower generation in ShanDong Province

Economic research on the value chain can be traced back to the 1980s. Professor Michael Porter of Harvard Business School first proposed the concept of “value chain” in his book “Competitive Advantages” in 1985. Porter believes that “the company’s value creation process is mainly through basic activities (including production, marketing, transportation and after-sales services, etc.)

and supporting activities (including raw material supply, technology, human resources and finance, etc.). Activities are interrelated in the process of company value creation, which constitutes the chain of behavior of company value creation, which is called the value chain. Porter also believes that not only the value chain exists within the company, but also the value chain of another company is connected with the value chain of other companies. The value chain of any company exists in a value system composed of many value chains, and each system in the system the link between value behaviors has a crucial impact on the size of a company’s competitive advantage. This view has made a groundbreaking contribution to the value chain doctrine.

The automotive industry has a very high degree of relevance, and the value created in each link of its value chain is different, and its upgrade path and upgrade space are different due to different chain drive and governance models. The objects selected in the past research institutes tend to focus on the passenger car manufacturing process in the automobile industry, and are separated from the background of the global slice transfer of the automobile industry. All along, China’s automobile development policy is biased towards the whole vehicle manufacturing process, and does not look at the development of the automobile industry from the perspective of the value chain. As a result, China’s current parts and components industry does not have a complete supporting capacity, and the development is backward, and zero. The backwardness of the components in turn restricts the development of China’s vehicle industry, which hinders the extension of China’s automobile industry to the high-end links of the value chain. Today, with the globalization of automobile production and sales and the full opening of China’s automobile market, how to effectively use foreign direct investment to improve the position of China’s automobile industry in the value chain has become an urgent problem to be solved.

**Figure 2:**
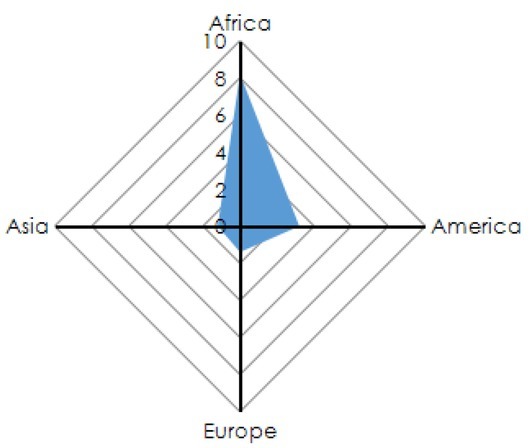
Global distribution of automobile sales in the world

In the process of industrialization of China, the automobile industry has already been the pillar industry of national economy. In the past 30 years, China’s automobile industry has implemented the policy of “gaining technologies at the expense of market” by substantially attracting foreign direct investment. The automobile industry has made great progress in terms of output scale and product structure, and the overall economic benefits have been greatly improved. The domestic automobile market is flourishing, new products are continuously launched and the quality is continuously improved, which basically meet the market demand at home and abroad. However, there are many problems in China’s auto industry, such as technology dependence, weak spare parts industry and lack of independent brand. In the past literature on automobile industry, the research objects often focus on the whole vehicle in the automobile industry, ignoring the background of the globalization of automobile value chain. Based on this, this study applies the theory of global value chain and refers to the value-added model of the automobile industry to study the impact of foreign direct investment on the value increment of the automobile industry in China with available data. The conclusion is that the effect of transnational direct investment on the value increment of the automobile industry in China is not significant, even with inhibitory effect on the R & D of products 4,5. The global share of the automobile market is shown in Figure 2.

## 3 The implementation of cognitive artificial neural networks algorithm

### 3.1 The Introduction to Fuzzy Theory

**Figure 3:**
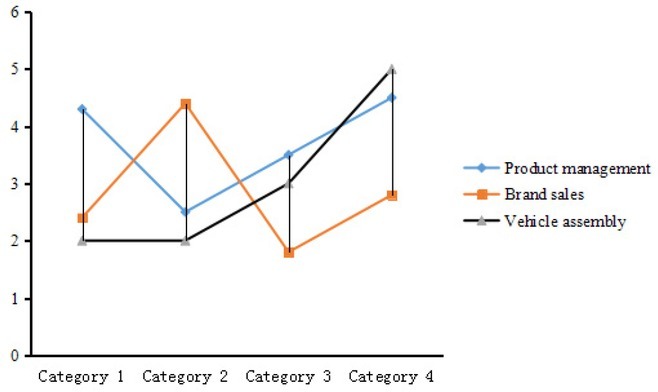
Value distribution curve of automobile industry chain

When some enterprises in the value chain carry out production according to the parameters (standards or rules) set by other subjects, the problem of governance arises. These parameters include what to produce (definition of products), how to produce (i.e. definition of producing process, including elements such as technique, quality, labor and environmental standards), when to produce, how much to produce, and price. On the left of point A is the production field, and on the right is the circulation field. Both fields have a circulation process, and the minimum value-added link is the link of the vehicle assembly, as shown in Figure 3.

### 3.2 Cognitive Artificial Neural Network

Kaplinsky & Morris (2001) talked about cluster upgrading from the perspective of value chain upgrading and thought there are mainly four types of cluster upgrading: process upgrading, production upgrading, functional upgrading and chain upgrading. Process upgrading refers to increasing input-output level by reorganizing product systems or introducing high technologies; production upgrading refers to the development of new products, adopting more complex product lines and improving quality faster than competitors; functional upgrading refers to accepting new functions or giving up old functions to improve skills; chain upgrading refers to shifting to new and relevant industrial value chains of high value (see Table 1) 6,7.

**Table 1:** The model of industry upgrading model under the framework of global value chain analysis

Product upgrade type	Product upgrading practice	Product upgrade performance
Technology upgrades	The production process is more efficient	Reduce costs and increase productivity
Product upgrade	New product development	New market share increased
Function upgrade	Better conditions for added value	Improve key functions in the value chain
The chain upgrade	Divestment of the original production and operation activities	The new product is differentiated

### 3.3 The integration of Fuzzy System and Cognitive Artificial Neural Network

In many links of a value chain, not every link creates equal value. The global economy is not bound by the standards and rules set by the free market alliances and intergovernmental organizations, but by the rules and standards set by the leading companies in the global value chain of the industry, and on this basis, the global value is proposed. The concept of chain governance: Governance of global value chains refers to the realization of non-market coordination between different economic activities and different links in the value chain through the relationship arrangement and institutional mechanism between companies in the global value chain.

**Figure 4:**
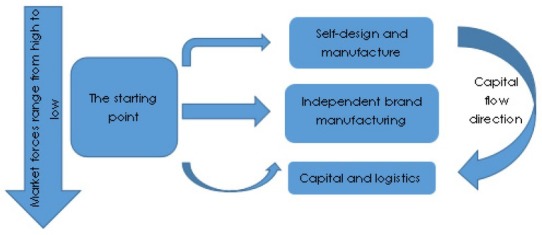
The upgrading approach of China’s automobile industry in the global value chain

One of the basic ideas in the study of global value chain is that among numerous value links

in the whole global value chain of a certain industry, it’s not every link that creates value, and according to the value-added capacity of each link, the whole value chain can be divided into several links or segments. In other words, the whole chain of values is characterized by a hierarchy of values [8]. The high value-added value link is generally the core link of the global value chain, and the global governance rules of the whole value chain are also determined by these core links. According to the level of value-added ability, there exists a strict hierarchy system of comparative advantages in different regions. The process of matching the value hierarchy system of global value chain with the global comparison hierarchy in different regions is the process of vertical separation and spatial reconstruction of value links in global value chain. The hierarchy system of local industrial clusters on the same value chain in the real world is determined by the hierarchy system of value links. According to geographic spatial relationship, it can be divided into global value chain, transnational value chain, domestic value chain and local value chain, as shown in Figure 4.

## 4 Example Analysis of Small Hydropower Generation Capacity Prediction

### 4.1 Cognitive Artificial Neural Networks algorithm

Humphrey and Schmitz’s method on value chain classification has increased the way that industrial clusters are embedded in the global value chain. Corresponding to the four types of their chain governance models, there are four embedding modes of local industrial clusters: bureaucracy embedding, which refers to one behavior subject is completely controlled by the other, or completely control the other, for example, being embedded into the global value chain by acquisition or being acquired; market

embedding, which refers to that the economic actors are embedded in the global value chain through the direct purchase and sale of various goods or services, and the parties are fully symmetric in power and only need to trade through a price mechanism, such as the direct sale of goods to overseas markets by means of international trade; network embedding, which refers to one actor is embedded in the global value chain with the complementary advantages needed by other actors, and all parties are symmetric in power, but often coordinate some activities by non-price mechanism; quasi-bureaucracy embedding, which refers to one actor becomes the dominant person in the global value chain by virtue of some advantages, while the other actors are in a subordinate position, such as embedding the global value chain through OEM, ODM, etc. and quasi-bureaucracy embedding includes two types of embedding such as buyer driving embedding and producer driving embedding.

Artificial neural network is a statistical tool that simulates human brain for thinking and learning. BP neural network is the most widely used artificial neural network. BP algorithm is a three-layer topology including input layer, output layer and hidden layer. There is a mentoring learning algorithm. The basic principle is error back propagation and information forward propagation. The operation process is: training the network with the sample, continuously changing the connection weight between the neurons, and finally making the difference between the expected output value and the actual output value within a preset error range, thereby stopping the training, and the process of storing the network connection weights and completing the training.

**Figure 5:**
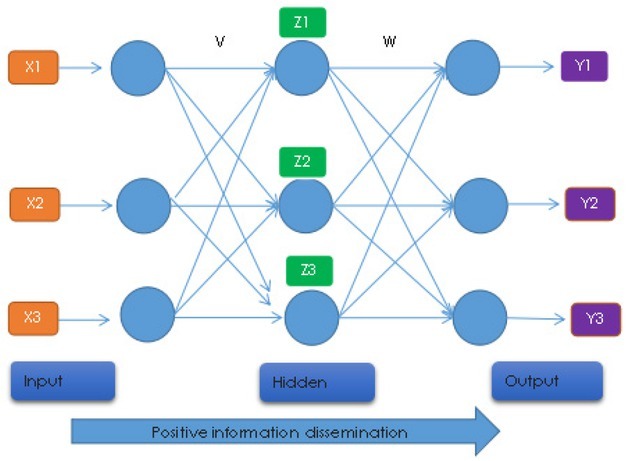
Cognitive Artificial Neural Networks algorithm

Back propagation is a multilayer feed forward network trained by error back propagation algorithm and is one of the most widely used neural network models. BP network can learn and store a large number of input-output pattern mapping without revealing the mathematical equation describing the mapping in advance. Its learning rule is to use the steepest descent method to adjust the weights and thresholds of the network continuously through the backward propagation, so as to minimize the sum of the square errors of the network. The model topology of BP neural network includes input layer, hidden layer and output layer. The cognitive artificial neural networks model is shown in Figure 5.

### 4.2 Simulation Calculation

Although the structure of the industry can be changed, it’s not good to be excessively dependent on changes in the structure of the industry to choose their new value chain and new customers before entering the industry. The change in the structure of the industry not only needs strong strength, but also works like a double-edged sword, since it can improve the profitability of the structure of the industry, but it can easily destroy this profitability. For example, a new product design that removes barriers to invasion or exacerbates competitiveness could destroy the industry’s foundation for long-term profitability, even though the promoters of the new product would temporarily make huge profits. In general, enterprises often neglect the long-term impact on the industry structure when they make strategic decisions, and they can only see the temporary improvement of their competitive position due to temporary success, but cannot foresee the reaction of competitors, which may have bad consequences. If main competitors follow suit, the profitability of the industry structure will be greatly weakened, so that all enterprises in the industry are suffering from it; however, even if all enterprises in the industry pursue a modest and passive market strategy, the competition in the industry will not necessarily be completely alleviated, because this will induce new competitors to enter, and then stir up competition. So, the more intense the competition in the industry is, the more helpful it can prevent new competitors from entering.

### 4.3 Data selection

**Figure 6:**
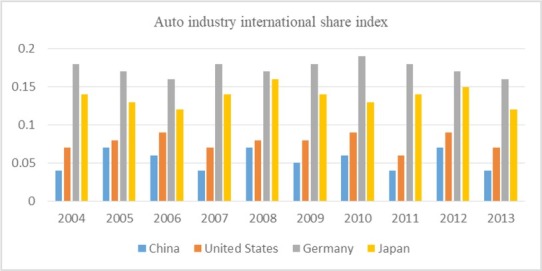
Comparison of Auto industry international share index from 2004 to 2013

Competitive advantage is the core of enterprise performance in competitive market. There are two basic forms of competitive advantage: cost leadership and differentiation. The competitive advantage comes from many separate activities carried out by enterprises in the process of design, production, marketing, delivery and other auxiliary processes. Each of these activities contributes to the relative cost position of the enterprise and lays the foundation for differentiation. The difference between competitors’ value chain is the key source of the competitive advantage. As a strategic tool, value chain plays an important role in analyzing relative cost position, differentiation and obtaining competitive advantage. Cultivating core competitiveness achieves sustainable development of enterprises. Through analyzing the relationship between the internal and external value chain of Laigang Group and the competitive advantage, we can see that Laigang Group has certain scale economic advantage in the industry, but it has no obvious advantage in variety, geographical location, resources and enterprise management. Therefore, Laigang Group should optimize the allocation of resources from a strategic perspective, foster the core competitiveness of enterprises, and achieve sustainable development. It should strengthen strategic management and carry out strategic positioning. In terms of overall competitive strategy, Laigang Group should mainly adhere to the leading cost, and strive to improve the differentiated advantages of such high value-added products as special steel, plates and strips. In order to integrate the cost advantage and the difference advantage, the whole value activity complex must be reconfigured. Table 2 shows the Auto Industry International Share Index from 2004 to 2013, Comparison of Auto industry international share index from 2004 to 2013 as shown in Figure 6.

**Table 2:** Auto industry international share index from 2004 to 2013

Year Country	2004	2005	2006	2007	2008	2009	2010	2011	2012	2013
China	0.04	0.07	0.06	0.04	0.07	0.05	0.06	0.04	0.07	0.04
United States	0.07	0.08	0.09	0.07	0.08	0.08	0.09	0.06	0.09	0.07
Germany	0.18	0.17	0.16	0.18	0.17	0.18	0.19	0.18	0.17	0.16
Japan	0.14	0.13	0.12	0.14	0.16	0.14	0.13	0.14	0.15	0.12

## 5 Conclusions

This study mainly combines the theory of global value chain to analyze the upgrading of China’s automobile industry cluster. First of all, it sorts out the theories of global value chain. Besides a brief summary of theories on global value chain in terms of driving mechanism, governance mode and upgrading path, this study also theoretically analyzes the fragmentation of global value chain and the formation of local industrial clusters, and points out that the fragmentation of global value chain promotes the formation of local industrial clusters. Secondly, it expounds the distribution of the value chain of the automobile industry cluster and the development of China’s automobile industry cluster and points out some problems existing in their development process. Thirdly, this study analyzes the status of China’s auto industry cluster, and it is considered that China’s automobile industry cluster is in a weaker position in the global value chain. This study proposes the path selection of upgrading for two kinds of different industrial clusters, mainly product upgrading or function upgrading. Finally, this study puts forward countermeasures of upgrading the automobile industry cluster in China.
